# Fabrication of Bacterial Cellulose-Curcumin Nanocomposite as a Novel Dressing for Partial Thickness Skin Burn

**DOI:** 10.3389/fbioe.2020.553037

**Published:** 2020-09-15

**Authors:** Wasim Sajjad, Feng He, Muhammad Wajid Ullah, Muhammad Ikram, Shahid Masood Shah, Romana Khan, Taous Khan, Ayesha Khalid, Guang Yang, Fazli Wahid

**Affiliations:** ^1^Department of Biomedical Sciences, Pak-Austria Fachhochschule Institute of Applied Sciences and Technology, Haripur, Pakistan; ^2^Hubei Key Laboratory of Economic Forest Germplasm Improvement and Resources Comprehensive Utilization, Huanggang Normal University, Huanggang, China; ^3^Department of Biomedical Engineering, Huazhong University of Science and Technology, Wuhan, China; ^4^Department of Pharmacy, COMSATS University Islamabad, Abbottabad, Pakistan; ^5^Department of Biotechnology, COMSATS University Islamabad, Abbottabad, Pakistan; ^6^Department of Environmental Sciences, COMSATS University Islamabad, Abbottabad, Pakistan

**Keywords:** bacterial cellulose, biocompatibility, burns, curcumin, tissue regeneration, wound healing

## Abstract

The current study aimed to fabricate curcumin-loaded bacterial cellulose (BC-Cur) nanocomposite as a potential wound dressing for partial thickness burns by utilizing the therapeutic features of curcumin and unique structural, physico-chemical, and biological features of bacterial cellulose (BC). Characterization analyses confirmed the successful impregnation of curcumin into the BC matrix. Biocompatibility studies showed the better attachment and proliferation of fibroblast cells on the BC-Cur nanocomposite. The antibacterial potential of curcumin was tested against *Escherichia coli* (*E. coli*), *Pseudomonas aeruginosa* (*P. aeruginosa*), *Salmonella typhimurium* (*S. typhimurium*), and *Staphylococcus aureus* (*S. aureus*). Wound healing analysis of partial-thickness burns in Balb^c^ mice showed an accelerated wound closure up to 64.25% after 15 days in the BC-Cur nanocomposite treated group. Histological studies showed healthy granulation tissues, fine re-epithelialization, vascularization, and resurfacing of wound bed in the BC-Cur nanocomposite group. These results indicate that combining BC with curcumin significantly improved the healing pattern. Thus, it can be concluded that the fabricated biomaterial could provide a base for the development of promising alternatives for the conventional dressing system in treating burns.

## Introduction

Skin, as a vital organ, prevents the water loss from the body and keeps the internal environment moist. It encounters acute or chronic injuries in case of burns, accidents, injuries, or microbial contamination, which cause wounds that subsequently require healing. Wound healing is a complex process, involving different events such as coagulation, inflammation, cell division and migration, the formation of connective tissues and blood vessels, development of extracellular matrix (ECM), and maturation of epithelium (Mohanty and Sahoo, 2017). The healing process is typically comprised of four highly arranged phases, including homeostasis, inflammation, proliferation, and remodeling (Azuma et al., 2015; Ho et al., 2017).

The conventional wound dressings such as cotton and impregnated gauzes are painful, less efficient, causing damage to the newly formed epithelial lining skin, and unable to maintain ideal conditions for tissue repair (Di et al., 2017). In addition, severe burn injuries left a lifetime social and psychological impacts on the victim due to post burns scars, physical disfigurement, and contractures. To date, extensive efforts have been made in burn therapeutics to improve the survival of victims; however, burn-related morbidity and mortality are still alarming issues (Coban, 2012). Therefore, further efforts are required to develop advanced biomaterials-based wound dressings and potential alternatives to traditional counterparts by using tissue engineering strategies which focus on synthesis or regeneration of tissues and amplifying it *in vivo* by using active biofactors and a three-dimensional biocompatible, biodegradable, and porous scaffolds (Duan and Wang, 2010; Aljohani et al., 2018; Torgbo and Sukyai, 2018; McCarthy et al., 2019a,b). An ideal 3D scaffold supports the cellular activities for easy adhesion, penetration, growth, proliferation, and differentiation, in addition to being non-toxic, biodegradable, mechanically stable, and permeable to diffusion of nutrients and oxygen flow (Hu et al., 2014; Douglass et al., 2018). These features bring forward three basic requirements for a wound dressing material: scaffold, bioactive entity, and active cells, in order to mimic the 3D architecture of ECM of native tissues (Oliveira Barud et al., 2015). A variety of dressing systems have been designed in the past decades for efficient healing of dermal wounds, which are mainly comprised of different polymers and a variety of natural products.

Curcumin, a turmeric derivative of the ginger family, is extracted from the dried rhizome (Shehzad et al., 2013). It contains different bioactive ingredients like demethoxycurcumin (curcumin II), bisdemethoxycurcumin (curcumin III), and a newly discovered cyclocurcumin, collectively known as curcuminoids. It possesses wound-healing capabilities, antioxidant, and antineoplastic and can effectively inhibit the growth of various infectious antibiotic-resistant pathogens (Krausz et al., 2015). However, its systematic administration in the therapeutic application has been limited by its low aqueous solubility, rapid metabolism, poor tissue absorption, and limited plasma (Mohanty and Sahoo, 2017). To date, different topical formulations of curcumin like hydrogels, films, emulsions, and nano-formulations have been prepared for its control and targeted delivery to the wounded area (Mohanty and Sahoo, 2017). These formulations are able to deliver the drug to the target site owing to their small size, which can effectively cross the skin (Flora et al., 2013).

Recently, biopolymers have received immense consideration for biomedical applications. To this end, the use of bacterial cellulose (BC), produced by microbial cells (Ullah et al., 2017) and cell-free systems (Ullah et al., 2015; Kim et al., 2019), has received a growing interest as a biomaterial in various biomedical applications (Klemm et al., 2001; Czaja et al., 2006; Khan et al., 2015a,b; Hussain et al., 2019), specialty membrane (Rajwade et al., 2015), biosensors (Jasim et al., 2017; Farooq et al., 2020), and drug release (Numata et al., 2015; Li et al., 2018). These applications utilize both pure BC and its composites with other materials, such as biopolymers including collagen (Takeda et al., 2016), silk-sericin (Lamboni et al., 2016), gelatin (Khan et al., 2018), alginate (Kirdponpattara et al., 2015), and chitosan (Ul-Islam et al., 2019), and nanoparticles such as silver (Maneerung et al., 2008), zinc (Ul-Islam et al., 2014; Khalid et al., 2017a), titanium dioxide (Khan et al., 2015a,b; Ullah et al., 2016a; Khalid et al., 2017b), and gold (Khan et al., 2018), as well as clay materials such as pristine and modified montmorillonite (Ul-Islam et al., 2013b). The unique features of BC include high purity, better mechanical properties, high water holding capacity (WHC) and slow water release rate (WRR), active surface area, moderate biocompatibility, biodegradability, three-dimensional (3D) reticulate fibrous structure, micro-porosity, optical transparency, and non-toxicity, and moldability into different shapes (Czaja et al., 2006; Ul-Islam et al., 2013a; Ullah et al., 2016b; Di et al., 2017). However, pristine BC lacks innate antibacterial and antioxidant properties (Ul-islam et al., 2020); therefore, fabrication or modification of BC with antimicrobial agents like nanoparticles could synergize its wound healing properties, thus obtaining a smart antimicrobial wound dressing system (Khalid et al. 2017a,b). The use of curcumin is quite well-known in traditional medicines for various skin wounds, particularly burns, cuts, and eczema (Khamrai et al., 2017). Several studies have demonstrated the potential of BC and curcumin for different biomedical applications: such as the use of modified BC-curcumin self-healing polyelectrolytic film (Khamrai et al., 2017), curcumin-loaded BC films for treating skin cancer (Subtaweesin et al., 2018), cellulose/curcumin composite films with antibacterial activity for food packaging applications (Luo et al., 2012), curcumin-loaded cellulose-halloysite nanotube composite hydrogel for anticancer and wound dressing applications (Huang et al., 2017), and cellulose/turmeric powder green composite films (Li et al., 2014). However, no such comprehensive study was conducted where the healing activity of these BC-curcumin nanocomposites was investigated for the partial-thickness burns.

The current study was aimed to fabricate curcumin-loaded bacterial cellulose (BC-Cur) nanocomposite as a potential wound dressing, with improved tissue regeneration and wound healing properties, for partial thickness skin burns by utilizing the inherent structural and biocompatible features of BC and therapeutic potential of curcumin. The fabricated BC-Cur nanomaterial was characterized for its structural and physico-chemical properties by different characterization techniques, while its potential wound healing activity was assessed in the burn animal model. The antibacterial activity of curcumin was tested against various burn wound pathogens. The curcumin showed remarkable antibacterial activity against the tested pathogens. The developed nanomaterial showed excellent wound healing and tissue regeneration potential, thus can be used as an efficient curcumin delivery system to the wounded area.

## Materials and Methods

### Materials

Glucose, sodium hydroxide (NaOH), and phosphate buffer saline (PBS) were purchased from Sigma Aldrich (St Louis, MO, United States). Nutrient agar and bacteriological peptone, citric acid, and yeast extract were supplied by the Oxoid Ltd., while dextrose by Daejung, South Korea. Curcumin and sodium dihydrogen phosphate (NaH_2_PO_4_) purchased from Merck & Company, Inc. (Darmstadt, Germany).

### Production and Processing of BC Sheets

Bacterial cellulose sheets were produced by *Gluconacetobacter xylinus* (*G. xylinus*: KCCM 40407) in Hestrin–Schramm (HS) medium following a previously reported protocol (Sajjad et al., 2019). First, the HS medium was prepared by dissolving the respective medium components in distilled water, and its pH was adjusted to 5.0 by using 0.1 M NaOH. Thereafter, the medium was sterilized by autoclaving at 121°C for 15 min at 15 psi. For the preparation of the pre-culture of *G. xylinus*, few bacterial colonies were picked from the culture plate and inoculated into 50 mL HS medium and incubated at 30°C in shaking incubator at 150 rpm for 20 h. For BC production, a 5% pre-culture was inoculated into the liquid HS medium and incubated at 30°C under static cultivation for 7 to 10 days. BC sheets, a hydrogel produced at the air-medium interface, were harvested and washed in running water to remove residual medium components. Thereafter, the washed BC sheets were treated in 0.3 M NaOH solution and sterilized by autoclave to kill any live cells and cell debris (Shah et al., 2010). The washed and sterilized BC sheets were preserved in distilled water at 4°C for further use.

#### Preparation of BC-Curcumin Nanocomposite

The BC-Cur nanocomposite was prepared by the *ex situ* method. Briefly, 1 wt.% (w/v) homogeneous aqueous suspension of curcumin was prepared through continuous stirring for 1 h. For the preparation of BC-Cur nanocomposite, 3 × 3 cm BC sheets were immersed in 100 mL of 1 wt.% curcumin suspension and placed in shaking incubator at 150 rpm for 24 h. The as-prepared nanocomposite was freeze-dried at −50°C for 10 h to obtain the BC-Cur nanocomposite membrane and stored in air-tight bags for characterization and further analysis.

#### Characterization of BC and BC-Cur Nanocomposite

The freeze-dried pristine BC and BC-Cur nanocomposite membranes were characterized for their structural and physico-chemical properties by using different techniques. The surface morphologies of both samples were determined by field emission scanning electron microscopy (FE-SEM) by fixing the samples on a brass holder followed by their coating with gold on a Cu SEM before analyzing it through FE-SEM (Nova NanoSEM450, FEI, Holland). Chemical structures of both samples were analyzed through Fourier-transform infrared (FTIR) spectroscopy by recording their spectra between 4000-500 cm^–1^ using an FTIR spectrophotometer (VERTEX 70, Germany). The polymorphic structures of samples were determined at the scanning angle between 0 and 40° using an X-ray diffractometer (X’Pert-APD PHILIPS, Netherlands), operated at X-ray generator tension and current values of was 40 kV and 30 mA, respectively. Further, the effect of curcumin impregnation on the crystallinity of BC was determined using below Eq. (1) (Ul-Islam et al., 2011):

(1) $$C\,r\,I=\left[\left\{I\,\left(200\right)-I\,\frac{a\,m}{I}\,\left(200\right)\right\}\times 100\right]$$

where CrI is the crystallinity index, while I (200) and I (am) represent the intensities of (200) plane and amorphous peaks, respectively.

#### Biocompatibility Assay

The biocompatibility assay was performed according to the established protocol by Khan et al. (2015a) with some modifications (Khan et al., 2015b). Briefly, the pristine BC and BC-Cur nanocomposite were briefly dipped in 70% ethanol followed by UV radiation in a clean bench for 1–2 h and finally washed 3 times with the Dulbecco’s phosphate-buffered saline (DPBS) for 5 min. Thereafter, both samples were incubated overnight at 37°C in Dulbecco’s Modified Eagle’s Medium (DMEM). Mouse fibroblast NIH 3T3 cells were detached from a previously cultured 100 mm plate through trypsin EDTA and seeded on BC and BC-Cur samples at a density of 3 × 10^5^ cells/mL and incubated for 1–5 days. The cell growth and spreading on the scaffold were monitored *via* a phase-contrast microscope. The cells were imaged at 1360 × 1024-pixel resolution on the scaffold at the respective time period by using a microscope (Olympus BX50, United States) equipped with a digital camera (Olympus DP70, United States).

#### Antibacterial Activity

The antibacterial activity of curcumin was examined *in vitro* against most common burn wound pathogens through well diffusion method (Sajjad et al., 2019). Three species of Gram-negative bacteria *Escherichia coli* (*E. coli*), *Pseudomonas aeruginosa* (*P. aeruginosa*), and *Salmonella typhimurium* (*S. typhimurium*) and one strain of Gram-positive *Staphylococcus aureus* (*S. aureus*) were selected for the current study. Silver sulfadiazine (SD) was used as a reference drug (positive control). The selected bacterial strains were grown on the nutrient agar growth medium. The wells of 6 mm diameter were made in the agar medium plates for curcumin suspension. A volume of 80 μl of an aqueous suspension of curcumin was added to each well. The inoculated dishes were incubated at 37°C for 24 h, and the zone of inhibition was measured to determine the antibacterial potential of the curcumin. The experiment was performed in triplicate for each strain. The percent inhibition was determined by using the below Eq. (2):

(2) $$P\,e\,r\,c\,e\,n\,t\,i\,n\,h\,i\,b\,i\,t\,i\,o\,n=\left(\frac{Z\,o\,n\,e\,o\,f\,i\,n\,h\,i\,b\,i\,t\,i\,o\,n\,t\,e\,s\,t\,s\,a\,m\,p\,l\,e\,s}{Z\,o\,n\,e\,o\,f\,i\,n\,h\,i\,b\,i\,t\,i\,o\,n\,o\,f\,s\,t\,a\,n\,d\,a\,r\,d}\right)\times 100$$

#### Wound Healing Studies in Burn Mice Model

BALB^c^ mice were kindly provided by the National Institute of Health, Islamabad, Pakistan, and used as an animal model. The mice were housed in polypropylene cages and had free access to a balanced diet and water. All animal experiments were performed in accordance with the regulation and recommendations of NIH 1985, 1996, and 2011 and the experimental procedures were approved by the “Research Ethics Committee” COMSATS (Commission on Science and Technology for Sustainable Development in the South) University Islamabad, Abbottabad Campus, Pakistan. For experimental analysis, mice were divided into four groups, including BC, BC-Cur nanocomposite, positive control, and negative control. A total of 20 animals were used in the experiment, with five animals in each group. The partial-thickness burns were inflicted according to the established protocol, established in our earlier studies (Khalid et al., 2017a; Sajjad et al., 2019). Before the induction of skin burn wounds, the mice were sedated through intraperitoneal injection of 100 mg.kg^–1^ ketamine and 10 mg.kg^–1^ xylazine. Hairs were removed from the selected area by using a disposable hair removing the blade, and partial thickness skin burns wounds were induced in the shaved area by perpendicularly applying a pre-flame heated metal bar (17 × 17 mm) with gravitational pressure for 7 s. Thereafter, 3 × 3 cm pieces of BC and BC-Cur nanocomposite were used to cover the skin burn wound area and stapled with disposable skin staplers (Ningbo Advan Electrical Co., Ltd., China) (Khalid et al., 2017a; Sajjad et al., 2019). As a positive control, 1 wt.% silver sulfadiazine was applied to burn wounds along with negative control (untreated mice). The healing process by BC-Cur nanocomposite was allowed continued for 15 days, while the bandage was changed on day 5 and 10 of treatment. The wound healing potential of BC-Cur nanocomposite was assessed by measuring the wounded area on day 0, 5, 10, and 15, and the healing percentage was determined in different mice groups by the below Eq. (3):

(3) Percent⁢healing=1-Wound⁢area⁢on⁢corresponding⁢dayWound⁢area⁢on⁢day⁢ 0×100

### Histological Studies

For histological studies, mice from all four groups were sacrificed on day 15 by using a cervical dislocation and tissues were surgically removed and stored in 10% formalin. For analysis, the tissues were first fixed in 15% formalin, followed by 15% alcoholic formaldehyde. Thereafter, tissues were dehydrated by treating with 70%, 80%, and absolute alcohol for 2, 3, and 6 h, respectively. For microscopic analysis, slides were prepared by clearing the tissues with xylene, embedding in paraffin wax, and finally cutting with a microtome before staining with hematoxylin and eosin (H&E) dye. The slides were observed under the microscope and imaged.

### Statistical Analysis

All data in the present study are expressed as mean and standard deviation. Statistical analysis was performed by using Student’s t-test using Sigma Plot. *p* ≤ 0.05 was considered statistically significant; asterisk (^∗^) represents significant statistical difference where ^∗∗∗^*p* ≤ 0.001, ^∗∗^*p* ≤ 0.01, and ^∗^*p* ≤ 0.05.

## Results and Discussion

### Production and Morphological Analysis of BC and BC-Cur Nanocomposite

**FIGURE 1:**
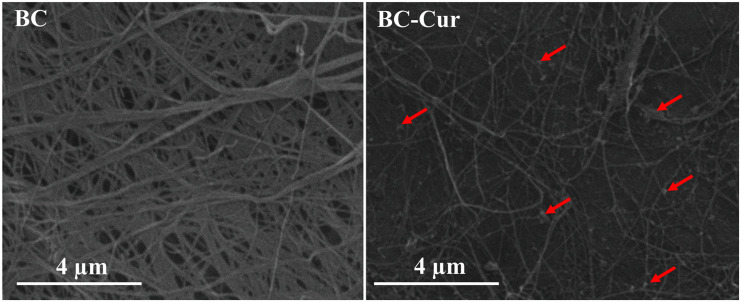
Field-emission scanning electron microscopic micrographs of BC and BC-Cur nanocomposite. The arrows indicate the presence of curcumin.

Bacterial cellulose is aerobically produced as a hydrogel membrane at the air-medium interface in static cultivation, whose thickness increases downward when new fibrils are added, and this process continues until all bacterial cells entrapped in the hydrogel membrane become inactive or die due to oxygen deficit (Shah et al., 2013). Being highly hydrophilic and porous in nature, the BC hydrogel effectively allowed the dispersion and impregnation of curcumin particles on the surface as well as into its matrix when immersed in 1 wt.% aqueous curcumin suspension as shown by the morphological analysis of BC-Cur nanocomposite through FE-SEM (indicated through arrows in Figure 1). The presence of OH functional groups vows BC with highly hydrophilic behavior, which results in the expansion of pore size when rehydrated, which facilitates the impregnation of other molecules such as polymer solution and micro- and nano-sized particles; curcumin powder in this case. The SEM micrograph showed a nearly uniform distribution of curcumin in the BC matrix (Figure 1).

### Structural Analysis of BC and BC-Cur Nanocomposite

**FIGURE 2:**
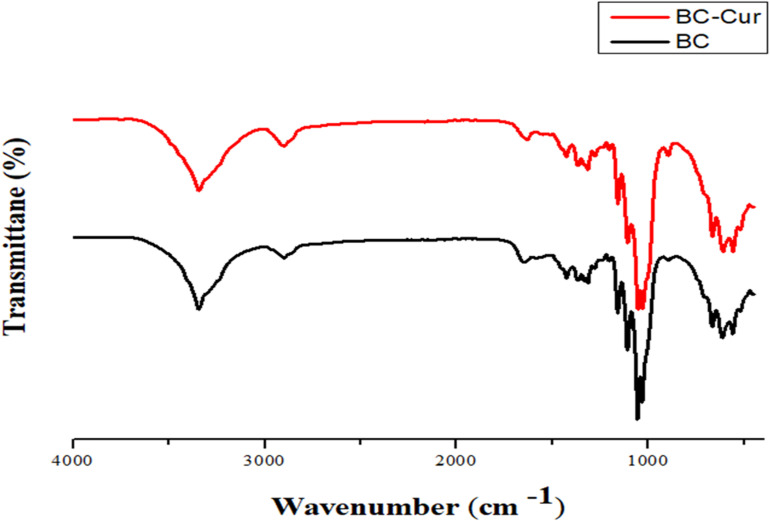
Fourier transform-infrared spectral analysis of BC and BC-Cur nanocomposite.

Fourier-transform infrared spectroscopy is used as an investigative tool to verify the presence of different functional groups and the nature of chemical interactions between such groups in the material under investigation. In the present study, FTIR spectroscopy of pristine BC and BC-Cur nanocomposite was carried out, and both spectra were comparatively analyzed to confirm the purity of BC and its possible interaction with curcumin. The combined spectra of pristine BC and BC-Cur nanocomposite is shown in Figure 2. FTIR spectrum of pure BC showed all characteristic peaks of pure cellulose, confirming the purity of synthesized BC by *G. xylinus* and the effectiveness of post-synthesis processing (i.e., NaOH treatment and washing with H_2_O). Specifically, the FTIR spectrum of pristine BC demonstrated characteristic peaks for − OH and − CH stretching at 3347 cm^–1^ and 2987 cm^–1^, respectively, while the presence of − CH group was further supported by the appearance of several small peaks between 1450–1200 cm^–1^. The peaks for symmetric deformation and bending vibration appeared at 1435 cm^–1^ and 1415 cm^–1^, respectively. A peak appeared at 1671 cm^–^1, which was attributed to the glucose carbonyl (−CO) group, while the peak for C-O-C stretching vibration appeared at 1055 cm^–1^. These characteristic peaks at respective positions confirmed the chemical structure of cellulose as well as purity of BC produced by *G. xylinus*, which is also in accordance with available literature (Ul-Islam et al., 2012; Ullah et al., 2016b). In contrast, the BC-Cur nanocomposite exhibited all characteristic peaks of BC; however, with a slight peak shift, which indicates an altered hydrogen-bonding pattern. For example, the peak for−CO group was slightly shifted from 1671 to 1663 cm^–1^, which could be attributed to the interaction of BC with curcumin. In addition to the slight peak shift observation, the spectrum of BC-Cur nanocomposite exhibited additional peaks around 1512–1518 cm^–1^ due to the aromatic skeletal vibrations of the benzene ring, which are in agreement with previous studies (Chidambaram and Krishnasamy, 2014; Subtaweesin et al., 2018). These observations not only confirmed the importation of curcumin into the BC matrix but also its chemical interaction with the cellulose fibrils.

**FIGURE 3:**
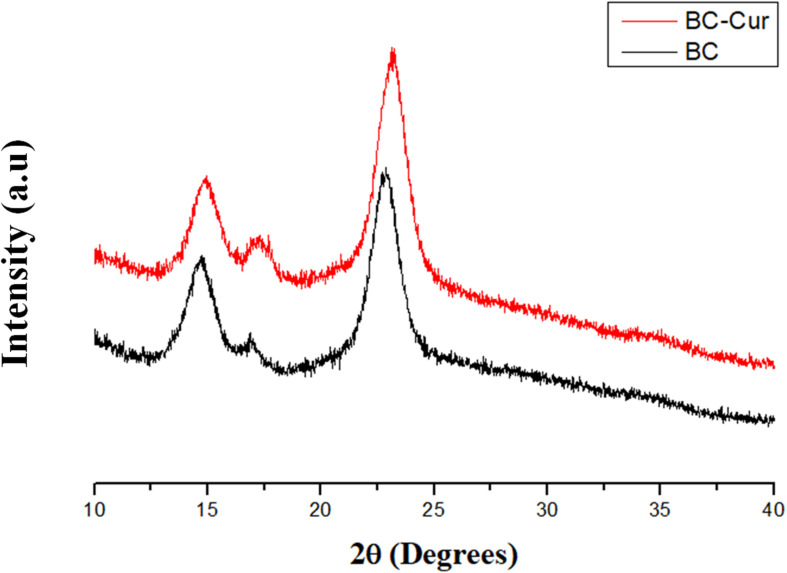
X-ray diffraction patterns of BC and BC-Cur nanocomposite.

Bacterial cellulose is a semi-crystalline material and possesses cellulose I and cellulose II polymorphic structures, when produced by microbial and cell-free systems, respectively (Ullah et al., 2016b). It contains three characteristic crystalline peaks when examined through XRD. Impregnation of any material into its matrix can potentially alter the crystal structure of BC, thus can lead to an increased or decreased crystallinity. XRD analyses of pristine BC and BC-Cur nanocomposite were carried out to investigate the possible effect of curcumin impregnation into the BC matrix. Comparative XRD spectra of the extended linear scanning (10–40°) of pristine BC and BC-Cur nanocomposite are shown in Figure 3. As expected, the XRD spectrum of pristine BC showed three characteristic peaks; including two distinct peaks at 2θ = 14.3° and 22.6° along a weak shoulder peak at 16.5°, corresponding to (1-1 0), (200), and (110) crystalline planes of cellulose I-β structure. These observations are in accordance with a well-characterized XRD spectrum of BC, as reported in available literature (French, 2014; Ul-Islam et al., 2014). Contrary, the XRD spectrum of BC-Cur nanocomposite showed similar peaks to pristine BC; however, with slight variation in the position and intensities of peaks. Specifically, the BC-Cur nanocomposite showed characteristic peaks at 2θ = 14.1°, 22.3°, and 16.2°corresponding to (1-1 0), (200), and (110) crystalline planes. The intensities of peaks at 2θ = 16.2° and 22.3° were slightly increased while that at 14.1° was slightly decreased as evident from Figure 3. These variations in peak intensity were quantitatively determined by measuring the degree of crystallinity of BC and BC-Cur nanocomposite from the relative integrated area of crystalline and amorphous peaks using Eq. 1. The relative crystallinities of BC and BC-Cur nanocomposite were 56.61 and 63.88%, respectively. The increased crystallinity of BC-Cur nanocomposite could be attributed to the crystalline nature of curcumin nanoparticles. As a matter of fact, the calculated crystallinity of any sample deviates to some extent from the actual value due to the presence of small crystalline peaks which raise the background and in turn contribute to increasing the amorphous and total area under consideration for calculating the crystallinity (French, 2014). Therefore, it is assumed that the calculated crystallinities of BC and BC-Cur nanocomposite might be slightly lower than the actual values.

### Biocompatibility of BC and BC-Cur Nanocomposite With NIH 3T3 Cells

**FIGURE 4:**
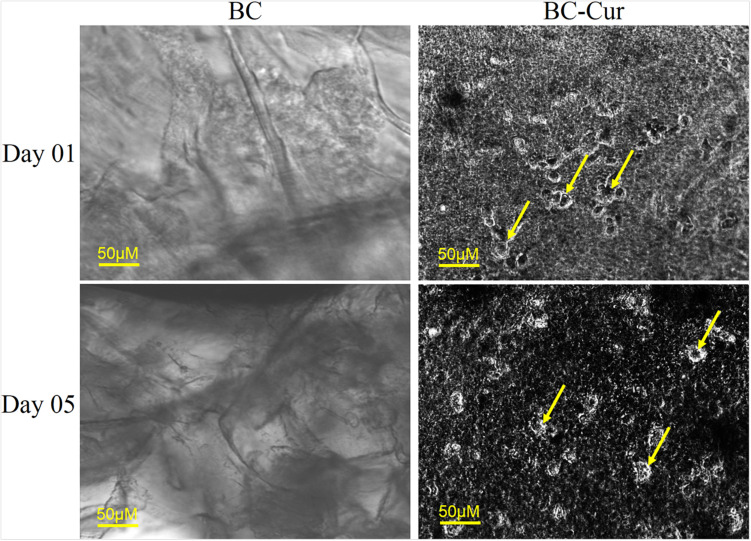
Cell adhesion and proliferation on BC and BC-Cur nanocomposite, 3T3 mouse fibroblast cells were cultured on BC and their composite with Cur showing cell growth on day 1 and 5. Arrows representing cells.

**FIGURE 5:**
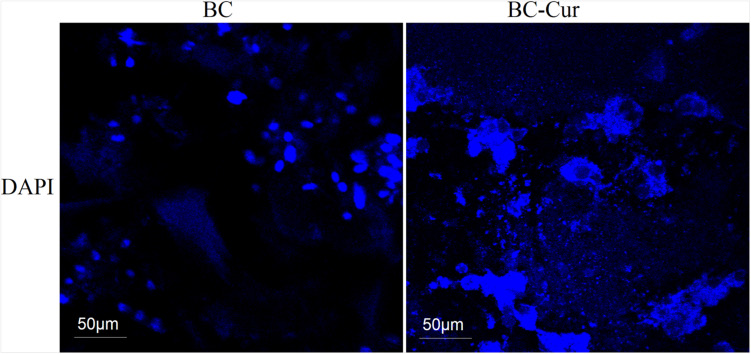
4′,6-diamidino-2-phenylindole (DAPI) staining of cells growth and attachment on BC and BC-Cur nanocomposites.

Curcuminoids are generally recognized as safe (GRAS) by the United States Food and Drug Administration (US-FDA). However, it may cause potential toxicity upon interaction with the plasma protein if excessively released into the blood stream. Therefore, in the current study, FDA-recommended safe concentration i.e., 1 wt.% of curcumin (Gupta et al., 2013; Dhakal et al., 2016) was used for the preparation of BC-Cur nanocomposite. *In vitro* biocompatibility of BC and BC-Cur nanocomposites were investigated using NIH 3T3 cells to evaluate their potential applications in wound healing. The mouse fibroblast cells were cultured on pristine BC and BC-Cur nanocomposites. In phase contrast, images were photographed on day 1 and 5. The results showed that there were few cells attached to the BC membrane surface (Figure 4). On the other hand, it can clearly be seen in images that more numbers of cells attached to the BC-Cur nanocomposite surface. In addition to increasing cell attachment, the BC-Cur nanocomposite showed distinguished behavior of the growth pattern as the cells appeared in the form of a colony or spheroids. For which further studies may need to elucidate the distinguished nature of BC-Cur nanocomposite. Furthermore, the cell attachment, spheroids formation, and spreading were confirmed by 4′,6-diamidino-2-phenylindole (DAPI) staining, as shown in Figure 5.

The SEM images showed a well porous morphology of BC and BC-Cur nanocomposite for potential cell adhesion and proliferation. Previously, it was observed that BC composites exhibited considerable cell adhesion and proliferation not only within the BC pores but also in its composites with other materials (Khan et al., 2016). The results showed that the addition of curcumin into BC improved the attachment and proliferation of NIH 3T3 cells, which is in accordance with a previous study, which reported that lower doses of curcumin enhance the cell growth and proliferation of fibroblast-derived from scar tissues (Kang et al., 2009). Another study reported that curcumin induces apoptosis of tumor cell lines; however, no apoptotic effect was observed on normal rat hepatocytes (Syng-ai et al., 2004). Thus, it can be concluded that the developed BC-Cur nanocomposite possesses a high level of biocompatibility, which makes it an ideal candidate for biomedical application, including skin tissue engineering.

### Antibacterial Activity

The antibacterial activity of curcumin was determined against the classic burn wound pathogens such as *E. coli, P. aeruginosa*, *S. typhimurium*, and *S. aureus*, by measuring the zone of inhibition and percent inhibition, and the results are summarized in Supplementary Table S1, and representative photographs are shown in Supplementary Figure S2. Curcumin is known for its antibacterial activity and produced inhibition zones of 15 ± 0 mm, 16.3 ± 0.4 mm, 16 ± 0 mm, and 15.5 ± 0.4 mm against *E. coli, P. aeruginosa*, *S. typhimurium*, and *S. aureus*, respectively (Supplementary Figure S2). Literature review shows that curcumin lyse the bacterial cell by causing damage to the cell membrane or through a specialized mechanism as a possible mode of action in *E. coli* (Wang et al., 2017). In another study, Wang et al. reported that curcumin shows its antibacterial activity against *S. aureus* by anchoring to the cell wall, break it, diffuse into the cell, and disrupt the structure of cellular organelles, and ultimately lead to apoptosis (Wang et al., 2017). Similarly, Yun and Lee (2016) in their study reported that curcumin induces the generation of reactive oxygen species (ROS), which accumulate inside the cell and damage the macromolecules, including proteins, nucleic acid (DNA/RNA), and lipid membranes, thus leading to apoptosis. These studies suggests that curcumin may induce the cell death through any of the above-described mechanisms. Overall, the findings suggest that the combination of BC and curcumin could be used as a smart antibacterial wound dressing system to treat the partial-thickness skin burn.

### *In vivo* Evaluation of Wound Healing in Burn Animal Model

**FIGURE 6:**
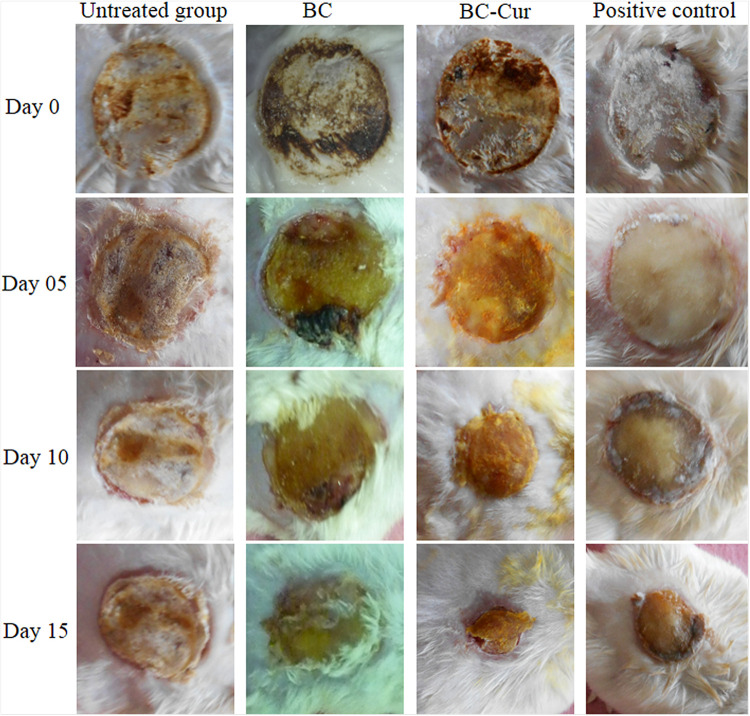
The photographs of the wound healing process in untreated, pristine BC, BC-Cur nanocomposite and positive control groups at day 0, 5, 10, and 15. Each photograph is a representative of three mice in each group.

In the present study, BC-Cur nanocomposite was developed as a wound dressing system and investigated its potential wound healing capability in the burn animal model. The results in Figure 6 show that the wound bed area started to contract on day 5 in each group and continued thereafter. Compared to BC, positive control, and untreated groups, the wound closing was much higher in the BC-Cur nanocomposite group. In comparison to the positive control group (silver sulfadiazine treated), the BC-Cur nanocomposite treated group showed more wound healing. The results of wound healing for all groups under investigation are summarized in Figure 6 and Table 1. Precisely, the wound area contraction in BC-Cur nanocomposite, pristine BC, positive control and negative control groups were 103.33 ± 12.24, 169 ± 13, 118.66 ± 13.14, and 202 ± 3.28, respectively, on day 15 (Table 1). Meanwhile, there was a significant difference in the percent wound healing of BC-Cur nanocomposite and other groups on day 15 of the experiment. The percent healing of the BC-Cur nanocomposite group was 64.25% in comparison to pristine BC, positive control, and the untreated group, which were found to be 41.60, 30, and 59%, respectively. These results indicate that the impregnation of curcumin into the BC matrix effectively enhanced its wound-healing capabilities. These observations are in agreement with previous studies reporting the therapeutic applications of BC and curcumin in wound healing. The nano-scale network structure of BC acts as a physical barrier to prevents the microorganisms from penetrating into the wound (Li et al., 2012a). El-Refaie et al. (2015) reported that curcumin-loaded gel-core hyaluosomes greatly enhanced the wound healing rate in a burn animal model. Similar results were shown by the curcumin-methoxy poly(ethylene glycol) (MPEG)-chitosan film in a rat model where the *N*,O-carboxymethyl chitosan-hydrogel effectively decreased the wound bed (Li et al., 2012b,a). Krausz et al., in their studies, demonstrated that curcumin nanocomposites inhibited the growth of burn-associated pathogens and enhanced wound healing (Krausz et al., 2015). It is reported that silver sulfadiazine, as a standard drug for burn wound treatment, cause delayed wound healing (Atiyeh et al., 2007) and significant cell impairment to human dermal fibroblast (Lee and Moon, 2003). In the current study, silver sulfadiazine showed less wound healing ability as compared to BC-Cur nanocomposite indicating a high wound healing potential of the developed nanocomposite, thus can find potential applications in skin burns.

**TABLE 1:** *In vivo* evaluation of wound healing in burn animal model.

	Average wound area (mm^2^)			
	
	Untreated group	BC	BC-Cur	Positive control
Day 0	289 ± 0	289 ± 0	289 ± 0	289 ± 0
Day 5	348.33 ± 20.2	318 ± 9.8	289 ± 14.2*	284.66 ± 19.8
Day 10	219 ± 29.1	200.33 ± 8	210 ± 14	241 ± 11.1
Day 15	202 ± 3.2	169 ± 13	103.33 ± 12.2***	118.66 ± 13.1
Healing (%) at day 15	30.10%	41.60%	64.25%	59%

The data represent the mean ± SD of three mice with percent healing. The statistical significance of wound reduction was evaluated at day 0, 5, 10, and 15 of post-wounding. p ≤ 0.05 was considered statistically significant (asterisk (*) represents significant statistical differences at p ≤ 0.001 ***, p ≤ 0.01 **, p ≤ 0.05 *, n = 3).

In conclusion, the results of the current study demonstrate that the *ex situ* developed BC-Cur nanocomposite exhibits significant wound healing potential for skin burns. Hence, nano-structured BC might be a promising carrier for curcumin to accelerate the wound healing process with the possibility of inhibiting the penetration of microbes, better patient compliance, minimizing the scar formation, and provide excellent recovery medium for complete skin regeneration.

### Histological Analysis

**FIGURE 7:**
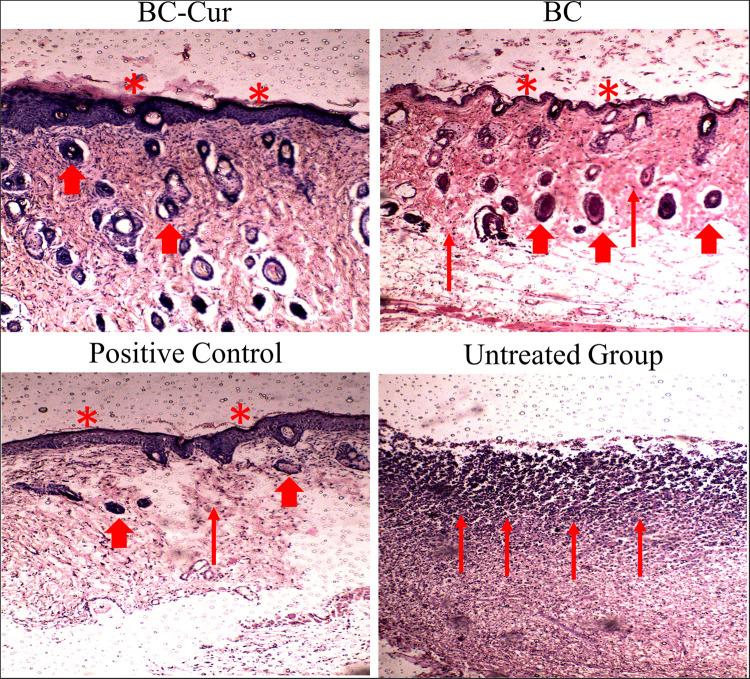
H&E stained sections of the wounds of BC-Cur, BC, positive control and untreated group (negative control) at day 15 post wounding. Thin arrow represents granulation, thick arrow shows hairs follicles and asterisk represents epithelization of epidermis layer of skin.

The tissue regeneration capabilities of BC and BC-Cur nanocomposite were assessed by histological analysis. For this purpose, the full thickness sections of both treated and untreated wounds were extracted on day 15 of the experiment and stained with H&E for microscopic examination of re-epithelialization, fibrogenesis, tissue regeneration, and formation of granulation tissues and the results are shown in Figure 7. The H&E stained microphotograph showed the migration of epithelium over the complete dermis, re-epithelialization, and granulation of tissues in the BC-Cur nanocomposite treated wound. Further, few inflammatory cells, with the absence of necrotic tissues and ulceration, were observed in the BC-Cur nanocomposite group (Figure 7), indicating its excellent wound healing and tissue regeneration capabilities. These results are in agreement with a previous study which demonstrated that curcumin administration increased the cell proliferation and collagen synthesis at the wound site and thus contributed to rapid wound healing in full-thickness excision wound model (Panchatcharam et al., 2006). On the other hand, the non-modified BC treated wound displayed mild inflammation, less ulceration, and absence of necrotic tissues in the H&E stained slide photograph (Figure 7). Moreover, the partial re-epithelialization, tissue regeneration, and presence of small granulation tissues indicate that the wound healing process was incomplete and still active. Histological analysis of both positive and negative controls was also performed to compare the wound healing, and tissue regeneration efficiencies of both BC and BC-Cur nanocomposite treated groups. The histological analysis of positive control presented a good wound healing capability; nevertheless, lower than BC-Cur nanocomposite treated group (Figure 7), which are also in agreement with *in vivo* evaluation of wound healing (Figure 6). While in the negative control group where animals were left untreated, a higher number of inflammatory cells, a dead slough of necrotic tissues, and ulceration were observed with no signs of re-epithelialization and tissue regeneration (Figure 7) which indicate that wound healing is still in an inflammatory phase. These results are summarized in Table 2. The results indicate that anti-inflammatory activity of curcumin and inherent wound healing properties of BC contributed to complete re-epithelialization, with well-formed differentiated epithelium and granulation tissue in the treated wounds. Therefore, these results of the efficient healing process in mouse models indicate the suitability of BC-Cur nanocomposite as a wound dressing system to treat partial-thickness skin burns.

**TABLE 2:** Histological evaluation of different wound healing events in untreated, pristine BC, BC-Cur nanocomposite, and positive control groups.

Group	Untreated	BC	BC-Cur	Positive control
Inflammation	+++	++	−	−
Necrotic tissues	++	−	−	−
Ulceration	++	+	−	−
Re-epithelialization	−	+	+++	++
Granulation tissues	−	+	+++	++
Healing	−	+	+++	++

*+++ (excellent); ++ (good); + (fair/mild); − (no).*

## Conclusion

Infectious burns are the most traumatic and challenging issue due to various pathological complications and microbial infections, which result in halted healing, scar formation, and uncontrolled necrosis of injured tissues. The *ex situ* modification allowed effective impregnation of curcumin into the BC matrix. FTIR spectroscopy confirmed chemical interaction between BC and curcumin. The addition of crystalline curcumin accounted for improved crystallinity of BC-Cur nanocomposite. The biocompatibility studies of the BC and BC-Cur nanocomposites confirmed their non-toxic nature for wound dressing application. The fabricated BC-Cur nanocomposite demonstrated accelerated wound healing, tissue regeneration, and wound contraction in a mouse burn model, thus showing the potential to be evaluated as a wound dressing material in treating partial thickness skin burns. The improved wound healing potential could be attributed to the additive effect of BC (inherently biocompatible) and curcumin (therapeutically active). These results suggest that the fabricated BC-Cur nanocomposite could be used as a potential topical antibacterial patch for wound healing and tissue regeneration in treating skin burns after further pre-clinical and clinical assessments.

## Data Availability Statement

The raw data supporting the conclusions of this article will be made available by the authors, without undue reservation.

## Ethics Statement

The animal study was reviewed and approved by Research Ethics Committee COMSATS University Islamabad, Abbottabad Campus, Pakistan.

## Author Contributions

FW and FH conceived the project, supervised the research, and wrote the manuscript. WS and AK designed and performed the experiments, analyzed the results, wrote the manuscript, and prepared the figures. MWU and GY performed the characterization and wrote the manuscript. MI and SMS assisted in biocompatibility studies. RK assisted in characterization analysis and manuscript writing. All authors read and approved the manuscript.

## Conflict of Interest

The authors declare that the research was conducted in the absence of any commercial or financial relationships that could be construed as a potential conflict of interest.
