# Supplementary material for: Fabrication of Bacterial Cellulose-Curcumin Nanocomposite as a Novel Dressing for Partial Thickness Skin Burn
Source: Front Bioeng Biotechnol. 2020 Sep 15;8:553037. doi: 10.3389/fbioe.2020.553037 (PMC7531241; doi:10.3389/fbioe.2020.553037)
Supplement: Supplementary file 1 [file Data_Sheet_1.docx]

Supplementary Material

**Fabrication of Bacterial Cellulose-Curcumin Nanocomposite as a Novel Dressing for Partial Thickness Skin Burn**

**Wasim Sajjad^1,5^, Feng He^2^*, Muhammad Wajid Ullah^3^, Muhammad Ikram^4^, Shahid Masood Shah^5^, Romana Khan^6^, Taous Khan^4^, Ayesha Khalid^5^, Guang Yang^3^, and Fazli Wahid^1^***

^1^Department of Biomedical Sciences, Pak-Austria Fachhochschule: Institute of Applied Sciences and Technology, Mang, Khanpur Road, Haripur, Pakistan

^2^Hubei Key Laboratory of Economic Forest Germplasm Improvement and Resources Comprehensive Utilization, Huanggang Normal University, Huanggang 43800, PR China

^3^Department of Biomedical Engineering, Huazhong University of Science and Technology, Wuhan 430074, PR China

^4^Department of Pharmacy, COMSATS University Islamabad, Abbottabad Campus 22060, Pakistan

^5^Department of Biotechnology, COMSATS University Islamabad, Abbottabad Campus 22060, Pakistan

^6^Department of Environmental Sciences, COMSATS University Islamabad, Abbottabad Campus 22060, Pakistan

***Correspondence**

**1. Fazli Wahid** (Email: [fazliwahid@cuiatd.edu.pk](mailto:fazliwahid@cuiatd.edu.pk))

2. Feng He (Email: [hfeng@hust.edu.cn](mailto:hfeng@hust.edu.cn))

Supplementary Material

## Supplementary Figures

##
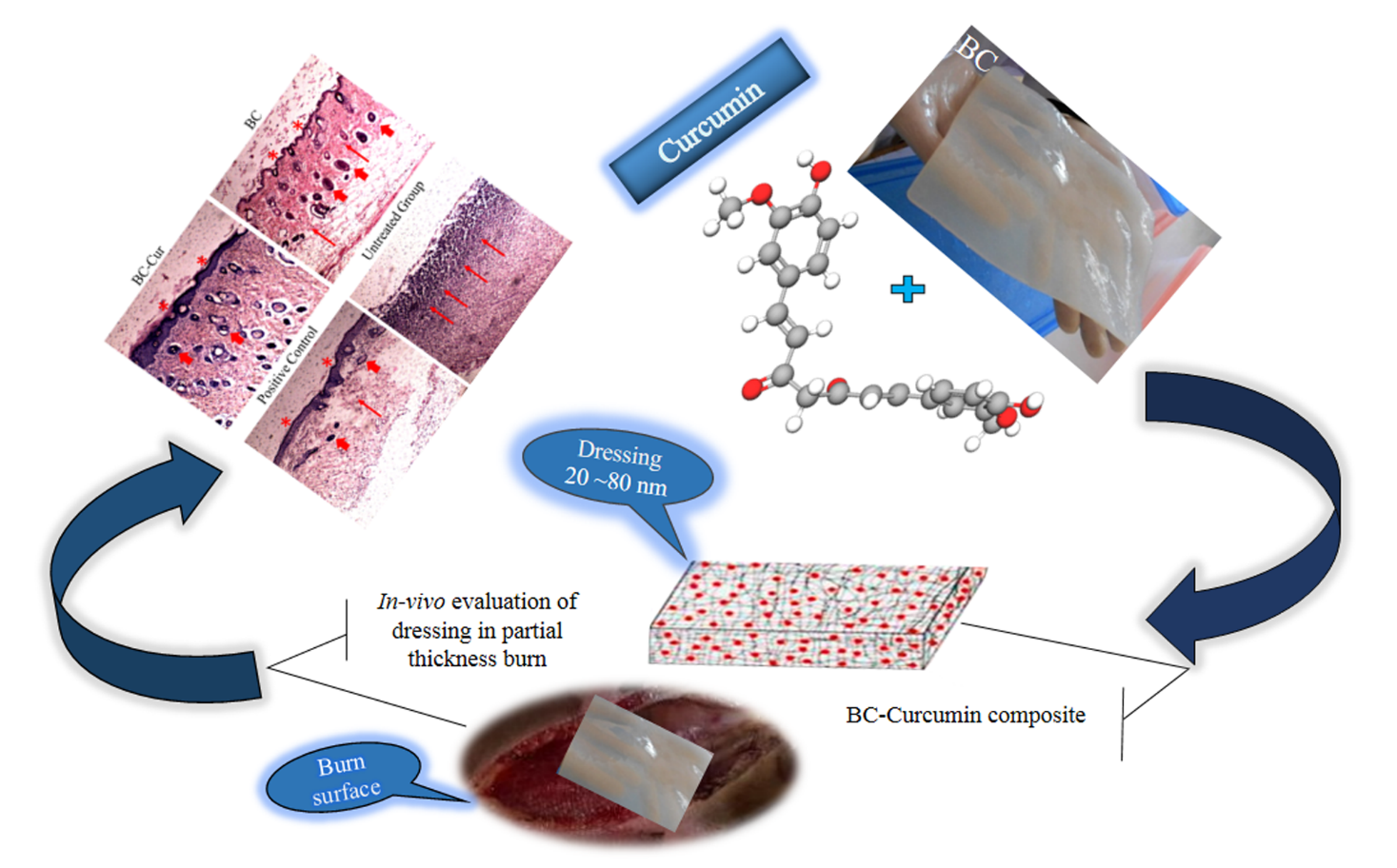


## Supplementary Figure 1. Graphical Abstract (Table of content)

**
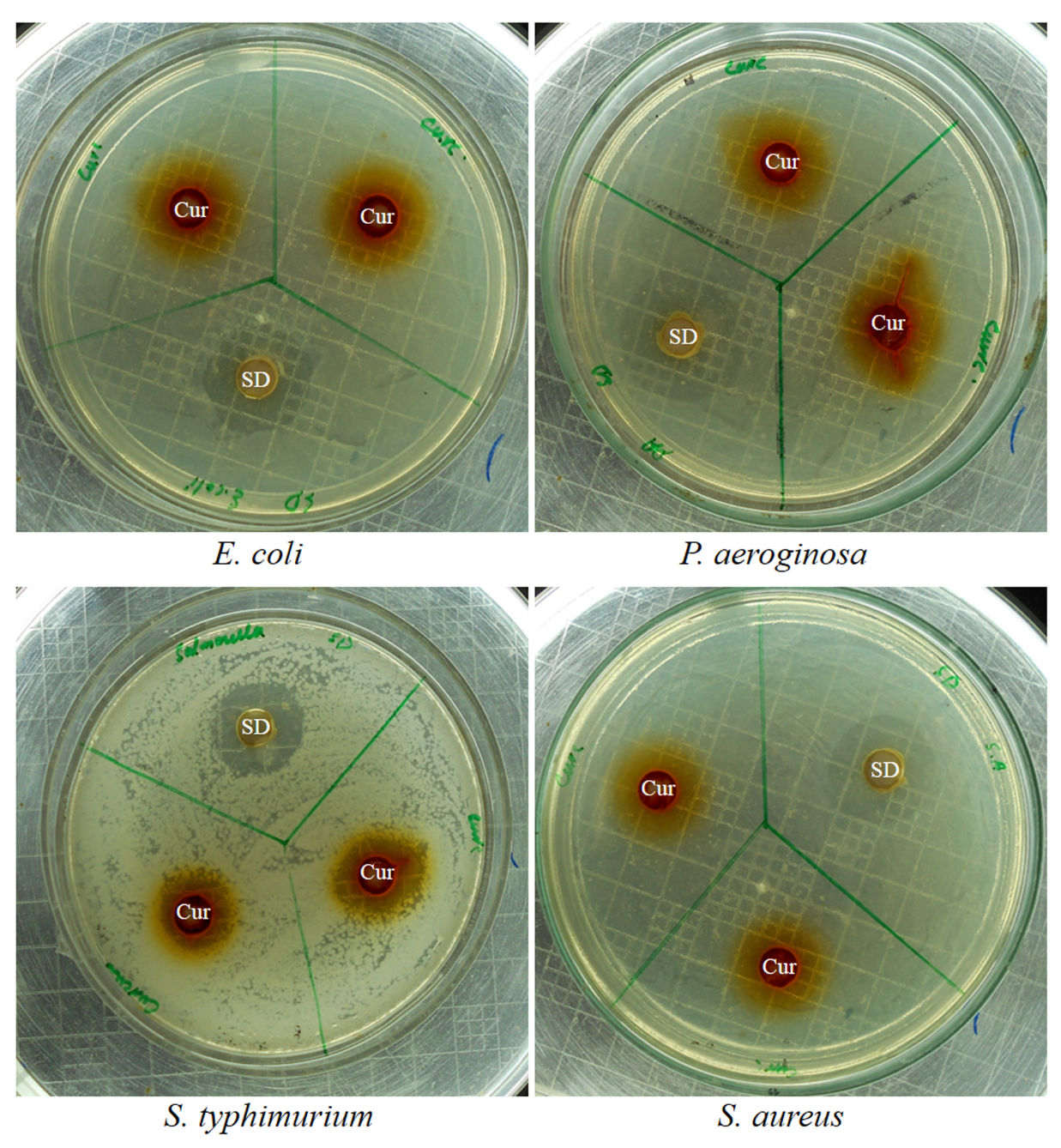
**

**Supplementary Figure 2.** Representative photographs of the antibacterial activity of the curcumin against burn wound pathogens. Curcumin showed prominent effects against *E. coli*, *P. aeruginosa*, *S. typhimurium* and *S. aureus*.

1. **Supplementary Tables**

**Supplementary Table 1.** The zone and percent inhibition of burn associated pathogens by curcumin in comparison to standard drug (silver sulfadiazine).

| **Bacterial species** | **Zone of inhibition (mm)** | | **Percent inhibition** |
| --- | --- | --- | --- |
|  | **Standard drug** | **Curcumin** |  |
| ***S. typhimurium*** | 18 ± 0 | 16 ± 0 | 82% |
| ***S. aureus*** | 17.6 ± 0.47 | 15.5 ± 0.4 | 81% |
| ***E. coli*** | 16 ± 0 | 15 ± 0 | 87% |
| ***P. aeruginosa*** | 18.3 ± 0.47 | 16.3 ± 0.4 | 82% |
